# Editorial

**DOI:** 10.1120/jacmp.v8i2.2694

**Published:** 2007-05-15

I hope you are enjoying the new look and feel of the JACMP. The transition to the latest version of software from the Public Knowledge Project was relatively painless, but not completely so. The Editorial Board (myself included) is still getting used to all the new features (and bugs), so please bear with us for the next few months.

We still have an open mind about how an open access online journal should function. When we moved to the PKP platform back in 2004, we took advantage of the opportunity for double‐blind peer review. For the most part, that has worked out well, and I have generally had positive feedback about the fairness of the JACMP review process. Moving to the future, one model that may be worth considering is to make the entire review process open access. In this model, de‐identified articles would be posted as soon as received, and anyone would have the opportunity to review the article anonymously for a time (say six weeks). Anyone posting a review would have the opportunity to rate the article. At the end of the review period, the article would be revised by the author, and the revision posted for ranking by the medical physics community. At the end of the ranking period, the top (say twelve) articles would be published and permanently archived. The others would be declined and eliminated from the system. This model eliminates the need for the traditional role of Associate Editors, except perhaps for the need to recruit reviewers if no one is willing to post a review for an article. If you have thoughts on this or other ideas on a beneficial publication model, please send me an email. It is clear to me that we have not seen the end of the changes that open access web technology will force on academia. Thanks for thinking about this.

Michael D. Mills, PhD

**Table:** 

**The Journal of Applied Clinical Medical Physics**	
**Editorial Board 2007**	
Editor‐in‐Chief	Michael Mills
Deputy Editor‐in‐Chief	Timothy Solberg
**Associate Editors**	
Radiation Oncology Physics	Nzhde Agazaryan, Salahuddin Ahmad John Antolak, Ivan Brezovich Jefferson Fairbanks, B. Gino Fallone Benjamin Nelms, Matthew Podgorsak Nikos Papanikolaou, William Salter Mehrdad Sarfaraz, Lu Wang Albert Zacarias
Medical Imaging	Geoffrey Clarke, William Pavlicek
Radiation Measurements	Larry DeWerd
Radiation Protection & Regulations	James Rodgers
Nonionizing Topics	Bhudatt Paliwal
Other Topics	Timothy Solberg
Book/Media Reviews	~
Editorials	~
**Journal Production Team**	
Proofreader	Heather Shand
Copy Editor	Ann Fothergill‐Brown
Layout Editor	Laura Shand
Manuscript Manager	Bonnie Ratcliff

